# Malignant Evaluation and Clinical Prognostic Values of m6A RNA Methylation Regulators in Glioblastoma

**DOI:** 10.3389/fonc.2020.00208

**Published:** 2020-03-09

**Authors:** Jianyang Du, Kuiyuan Hou, Shan Mi, Hang Ji, Shuai Ma, Yixu Ba, Shaoshan Hu, Rui Xie, Lei Chen

**Affiliations:** ^1^Department of Neurosurgery, The Second Affiliated Hospital of Harbin Medical University, Harbin, China; ^2^Translational Medicine Research and Cooperation Center of Northern China, Heilongjiang Academy of Medical Sciences, Harbin, China; ^3^Department of Digestive Internal Medicine, Harbin Medical University Cancer Hospital, Harbin, China

**Keywords:** glioblastoma, m6A RNA methylation, TCGA, methylation regulator, CGGA, prognostic signature

## Abstract

N6-methyladenosine (m6A) RNA methylation, the most common form of mRNA modification and regulated by the m6A RNA methylation regulators (“writers,” “erasers,” and “readers”), has been reported to be associated with the progression of the malignant tumor. However, its role in glioblastoma (GBM) has been poorly known. This study aimed to identify the expression, potential functions, and prognostic values of m6A RNA methylation regulators in GBM. Here, we revealed that the 13 central m6A RNA methylation regulators were firmly related to the clinical and molecular phenotype of GBM. Taking advantage of consensus cluster analysis, we obtained two categories of GBM samples and found malignancy-related processes of m6A methylation regulators and compounds that specifically targeted the malignant processes. Besides, we also obtained a list of genes with poor prognosis in GBM. Finally, we derived a risk-gene signature with three selected m6A RNA methylation regulators, which allowed us to extend the in-depth study and dichotomized the OS of patients with GBM into high- and low-risk subgroups. Notably, this risk-gene signature could be used as independent prognostic markers and accurate clinicopathological parameter predictors. In conclusion, m6A RNA methylation regulators are a type of vital participant in the malignant progression of GBM, with a critical potential in the prognostic stratification and treatment strategies of GBM.

## Introduction

Given the critical role of epigenetic regulation of DNA and histone (proteins) methylation in the underlying biological processes of mammals, the methyladenosine (mA) chemical modification of RNA may also be used as an novel epigenetic marker of far-reaching biological significance (1, 2). N6-methyladenosine (m6A) is the most prevalent internal chemical modification of mRNAs in higher eukaryotes, at a frequency of approximately three sites per mRNA (3–5). The m6A marks on mRNAs, similar to DNA and protein modification, are reversibly and dynamically regulated by methyltransferases (“writers”), binding proteins (“readers”), and demethylases (“erasers”). Six proteins have been identified in the m6A “writers” complex, including methyltransferase like 3 (METTL3) (6), methyltransferase like 14 (METTL14) (7), WT1-associated protein (WTAP) (8), RNA-binding motif protein 15/15B (RBM15/15B) (9), Vir like m6A methyltransferase associated VIRMA (also named as KIAA1429) (10), and zinc finger CCCH domain-containing protein 13 (ZC3H13) (11), which can catalyze the formation of m6A. As a function to decode m6A methylation and produce functional signals, the “readers” include YT521-B homology (YTH) domain-containing proteins (YTHDC1, YTHDC2) (12), YTH N6-methyladenosine RNA-binding proteins (YTHDF1, YTHDF2) (13), and heterogeneous nuclear ribonucleoprotein (HNRNP) protein families (14). “Erasers” are capable of removing the methyl code from target mRNAs, including fat mass and obesity-associated protein (FTO) (15, 16) and alkB homolog 5 (ALKBH5) (17).

The vital functions of RNA modification in processes of life have caught people's eyes in recent years. Substantial progress in regulating RNA transcription (18, 19), the event of processing (20, 21), splicing (5, 22), RNA stabilities (23, 24), and translation (25, 26) was witnessed in m6A post-transcriptional modifications. However, to date, the functions of the majority of RNA modifications found in mRNAs need further exploration. Notably, the functional roles of m6A methylation in tumorigenesis, tumor differentiation (27), proliferation (28), and invasion (27) remain elusive.

GBM is the most common and devastating primary tumor in the brain. Even the combined surgical resection, radiation therapy, chemotherapy, and other therapies were broadly used, the recurrence of the patients with GBM is inevitable. Besides, the median survival of GBM patients is <15 months after a definite diagnosis (29–31). The m6A RNA methylation regulators were also reported to be associated with self-renewal, radio resistance, and tumorigenesis of GBM stem cells (32). However, there is no comprehensive investigation of the expression of m6A RNA methylation regulators in GBM.

In the current study, the 13 m6A RNA regulators, which have been widely reported, were systematically analyzed using the GBM RNA sequencing data from The Cancer Genome Atlas (TCGA) (*n* = 174) and Chinese Glioma Genome Atlas (CGGA) (*n* = 249) databases. Taking advantage of m6A RNA methylation regulator-based consensus clustering analysis, we demonstrated the malignant process and obtained a list of genes with poor prognosis in patients with GBM. Importantly, we further validated these genes in the CGGA database and identified potential drugs targeting the malignant process of GBM using the Connectivity Map (CMap) (33). Besides, the risk-gene signature-derived from m6A RNA methylation regulators might be used as a novel biomarker that could identify GBM patients' prognosis and predict the clinicopathological parameters of GBM.

## Materials and Methods

### Data Acquisition

The RNA-seq transcriptome data and corresponding clinicopathological parameters of GBM patients were obtained from the TCGA database (http://cancergenome.nih.gov/) and the CGGA database (http://www.cgga.org.cn). The RNA-seq transcriptome data of healthy human tissue was obtained from the Genotype-Tissue Expression (GTEx) database (http://commonfund.nih.gov/GTEx/). We combined GTEx and CGGA data, and then harmonized them using quantile normalization and svaseq-based batch effect removal (34). The clinicopathological parameters for the CGGA and TCGA datasets are summarized in Table S1.

### Selection of m6A RNA Methylation Regulators

Thirteen widely recognized m6A RNA methylation regulators were retrieved from published literature. We then systematically compared the correlation between the expression of these m6A RNA methylation regulators and clinicopathological parameters in GBM patients.

### Bioinformatic Analysis

To further explore the role of m6A RNA methylation regulators in GBM patients, we clustered the GBM patients into two clusters by using the R package ConsensusClusterPlus (35). Heatmaps were drawn based on the average linkage method and the Pearson distance measurement method. Principal Component Analysis (PCA) was carried out by an R package called PCA to observe the distribution of gene expression in two clusters. Differential analyses for each gene in the pre-classified samples performed using the limma package in R (36). Fold change (FC) > 2 and adjusted *p*-value (*q*-value) < 0.01 were set as the cutoff values to screen for differentially expressed genes (DEGs). Gene Ontology (GO) functional analyses and Kyoto Encyclopedia of Genes and Genomes (KEGG) pathway enrichment analyses were performed to analyze the upregulated DEGs. The relationship between DEGs gene expression levels and overall patient survival time was illustrated by generating Kaplan-Meier plots. The correlation was tested using a log-rank test. Gene Set Enrichment Analysis (GSEA) was used to investigate the functions correlated with different clusters of GBM. |NES| > 1, adjusted *p* < 0.05, and FDR *q* < 0.25 were considered as statistically significant as described in a previous study (37).

### Construction of Protein-Protein Interactions (PPI) Network

PPI among selected genes was analyzed using the STRING database (38) and reprocessing via Cytoscape software (39). For better visualization, the color of the node in the PPI network was applied to reflect the logFC value, and the size of the node was applied to indicate the number of source proteins with the target protein. Molecular COmplex Detection (MCODE) (version 1.4.3), which is clustered based on given network topology, was used to discover densely connected regions. Then, the most significant module was filtered out by MCODE from the PPI networks. The criteria for selection were as follows: MCODE scores > 5, degree cut-off = 2, node score cut-off = 0.2, Max depth = 100, and *k*-score = 2.

### Construction of Gene-Signature

Univariate Cox regression analysis of the expression of 13 m6A RNA methylation regulators was conducted to determine the candidate genes associated with overall survival (OS). After that, an L1-penalized (LASSO) was performed to further identify the selected genes with independent prognostic value (40, 41). Finally, their regression coefficients were determined by the minimum criteria. The risk score for the signature was calculated accurately by the formula:

$$\begin{array}{l} \text{Risk score}=\sum_{i=1}^{n}\text{Coefi}*\text{xi}, \end{array}$$

where Coefi is the regression coefficient and xi is the expression of each selected gene. GBM patients were divided into low- and high-risk subgroups according to the median risk score. Kaplan–Meier plot was performed to compare the OS between two risk subgroups.

### Identification of Potential Compounds Targeting the Malignancy-Related Pathways

CMap (updated in September 2017) (https://clue.io/), as the world's largest perturbation-driven gene expression dataset, was employed to search for candidate chemical compounds that might target GBM stemness related pathways (33). The compounds were discovered by interrogating the CMap database of signatures with a query (a list of DEGs relevant to biological features of interest). The final results involved a CMap connectivity score (from −1 to 1) that indicated the degree of specificity associated with our particular query. 300 DEGs (150 downregulated and 150 upregulated) were selected for query methodology. Noteworthily, the closer the connectivity score of a compound was to −1, the more likely it was to reverse the genetic pattern we are querying. Finally, the compounds with the absolute value of CMap connectivity score of 90 or higher were considered to be potential therapeutic agents for functional validation.

### Statistical Analysis

Chi-square tests were used to compare the expression levels in GBM for age, gender, healthy samples, primary GBM and recurrence GBM, isocitrate dehydrogenase (IDH) status, and cytosine-phosphate-guanine island methylator phenotype (G-CIMP) status. One-way ANOVA was used to compare the distribution of the subtype of GBM (Classical, Mesenchymal, Neural, Proneural) (42). To evaluate the prediction accuracy of the risk score model, we performed a receiver operating characteristic (ROC) curve and calculated the area under the curve (AUC). Potential prognostic factors such as age (≤ 65 vs. > 65), gender (female vs. male), GBM subtype, and risk score (low-risk vs. high-risk) were analyzed by Univariate and multivariate Cox hazard regression.

## Results

### Expression Patterns of m6A RNA Methylation Regulators in GBM

**Figure 1:**
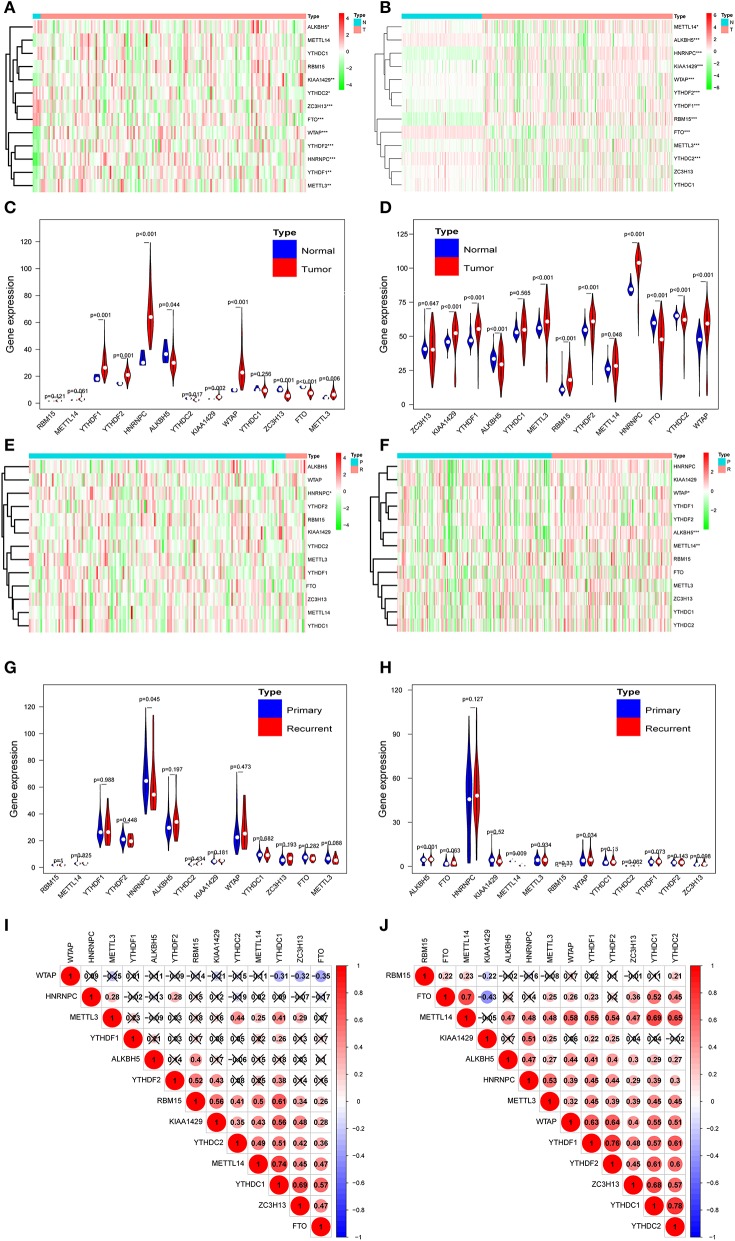
Expression of m6A RNA methylation regulators in normal, primary, and recurrence GBM. **(A,B)** Heatmaps of expression levels of the m6A RNA methylation regulators (normal sample vs. tumor sample) from TCGA database **(A)** and CGGA database **(B)**. **(C,D)** Violin diagrams corresponding to **(A,B)**. **(E,F)** Heatmaps of the expression levels of the m6A RNAVmethylation regulators (primary tumor sample vs. recurrent tumor sample) from TCGA database **(E)** and CGGA database **(F)**. **(G,H)** Violin diagrams corresponding to **(E,F)**. **(I,J)** Spearman correlation analysis of the 13 m6A regulators from the TCGA database **(I)** and CGGA database **(J)**. *P < 0.05; **P < 0.01; and ***P < 0.001; N, normal sample; T, tumor sample; P, primary tumor; R, recurrence tumor.

According to the essential biological functions of methylation regulators in the development of GBM, we first analyzed the relationship between each m6A RNA methylation regulator and the clinical molecular phenotype of GBM. The expression level of individual m6A RNA methylation regulator and different types of samples was presented as a heatmap. The result strongly indicates that the expression of the majority of m6A RNA methylation regulators was associated with the occurrence of GBM (Figure 1A). Importantly, the significant correlation between the occurrence of GBM and the expression levels of ALKBH5, METTL3, KIAA1429, HNRNPC, WATP, YTHDC2, YTHDF1, YTHDF2, and FTO were confirmed by the quantitative analysis of CGGA (Figure 1B). Compared with the healthy samples, the expression of METTL3, HNRNPC, WTAP, KIAA1429, YTHDF2, and YTHDF1 was upregulated, while the expression of ALKBH5, YTHDC2, and FTO was downregulated in the GBM samples (Figures 1C,D). Correlation analysis was also employed to investigate the relationship between the expression level of m6A RNA methylation regulators and the different stages (primary tumor stage and recurrent tumor stage) of GBM. Among the 13 m6A RNA methylation regulators, only HNRNPC was significantly related to the cancer recurrence (Figure 1E). Considering the dramatically imbalanced numbers of primary GBM (*n* = 156) and recurrent GBM (*n* = 13) in the TCGA database, the results of TCGA database analysis were not necessarily as accurate as those of CGGA, while the numbers of primary GBM (*n* = 140) and recurrent GBM (*n* = 109) were relatively balanced. Therefore, we analyzed the expression profile of the primary and recurrent GBM samples from the CGGA database and found that HNRNPC exhibited no correlation. Interestingly, as shown in Figure 1F, the expression of WTAP, ALKBH5, and METTL14 was significantly associated with the recurrence of GBM. These results suggested that WTAP, ALKBH5, and METTL14 were firmly related to the recurrence process of GBM (Figures 1G,H). We further explored the relationship between the expression of m6A RNA methylation regulators and GBM molecular subtypes. Notably, every expression of the majority of m6A RNA methylation regulators was associated with the subtype of GBM, except YTDHF2 and HNRNPC (Figure S1A). We also investigated the relationship between IDH status, G-CIMP status, and expression levels of each m6A RNA methylation regulator in GBM. The results revealed that the expression levels of METTL14, KIAA1429, YTHDC1, ZC3H13, and FTO were significantly dysregulated between different IDH status in the TCGA dataset (Figure S1B). As for the G-CIMP status, the expression of RBM15, YTHDF2, KIAA1429, and YTHDC1 exhibited a significant difference between G-CIMP+ and G-CIMP– (Figure S1C). We speculate that the change in the correlation of m6A RNA methylation regulators may be an internal characteristic that can reflect the external differences. From Figures 1I,J, different degrees of the relationship were observed between different m6A RNA methylation regulators. Most of the relationships between the regulators were positive correlations, especially YTHDC1, which contained the most active correlation with other regulators (Figures 1I,J).

### Identification of Two Clusters of GBM Samples With Different Clinical Characteristics

**Figure 2:**
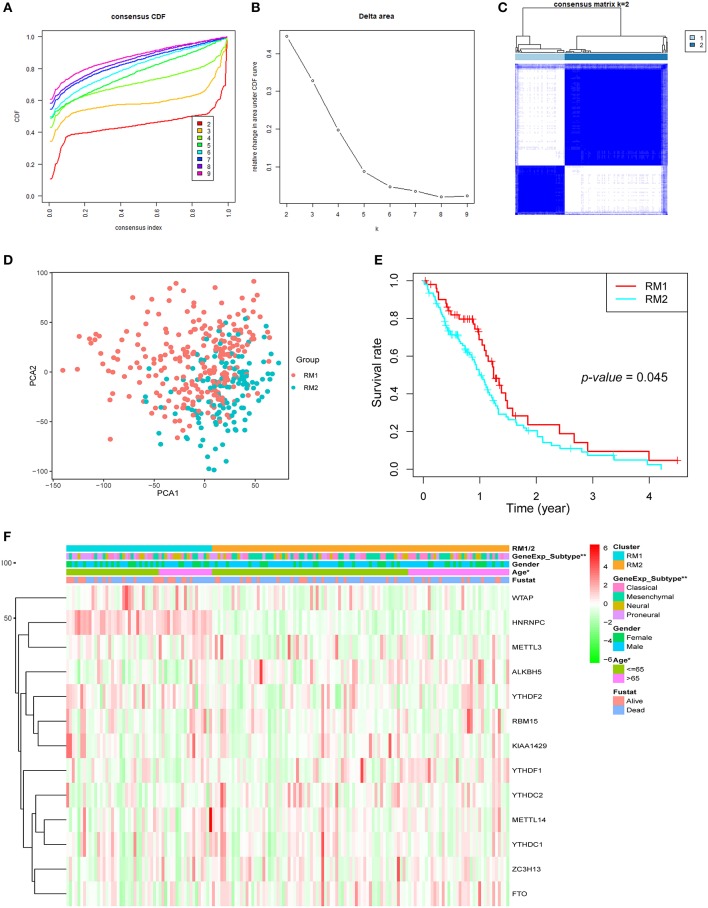
Differential clinicopathological features and overall survival (OS) of GBM in the RM1/2 subgroups. **(A)** Consensus clustering cumulative distribution function (CDF) for *k* = 2–10. **(B)** Relative change in area under CDF curve for *k* = 2–10. **(C)** Consensus clustering matrix for *k* = 2. **(D)** Principal component analysis of the total mRNA expression profile in the TCGA dataset. GBM patients in the RM1 subgroup are marked with red, GBM patients in RM2 are marked with green. **(E)** Kaplan–Meier OS curves for different subgroups. **(F)** Heatmap and clinicopathologic features of the two clusters (RM1/2) defined by the m6A RNA methylation regulators consensus expression.

Next, GBM samples with complete clinical parameters were selected for the subsequent consensus clustering analysis. From the view of the number of samples per group, an unbalanced distribution was observed in the three groups when *k* = 3 (Figure S2A). Hence, based on the expression similarity of the 13 regulators, *k* = 2 was the most optimum with clustering stability datasets increasing from *k* = 2–10 (Figures 2A–C and Figure S2). Then, GBM samples from the TCGA dataset were pre-classified into two groups (52 samples in one group labeled as RM1 and 106 samples in another group labeled as RM2) through consensus cluster analysis. The clinical features of the two groups are summarized in Table S2. The heatmap of cluster analysis showed that the 13 regulators could distinguish different samples, and the samples in the same cluster possessed a high correlation (Figure 2C). Principal component analysis was performed to elucidate the difference in transcriptional profiles between the RM1 and RM2 subgroups. The results investigated that there was a clear distinction between these two subgroups (Figure 2D). The survival curve according to Kaplan–Meier survival analysis for the clustered samples revealed a noticeable decrease in the OS in the RM2 subgroup compared with the RM1 subgroup, suggesting that the 13 methylation regulators could classify the GBM samples in prognostic level (Figure 2E). We further found that the median survival of the RM1 group was 1.4 years, while the RM2 was only 1 year. In addition, the clinicopathological features of these two subgroups were compared. The RM1 subgroup was markedly correlated with younger age at diagnosis (*P* < 0.05), Neural or proneural subtypes (*P* < 0.001), and G-CIMP– status (Figure 2F). The RM2 subgroup mainly contained GBM with older age at diagnosis, classical or mesenchymal subtypes, and G-CIMP+ status. Consistent with the report that classical and mesenchymal were more malignant compared to neural and proneural (42).

### Functional Annotation of Classification Determined by Consensus Clustering Analysis

**Figure 3:**
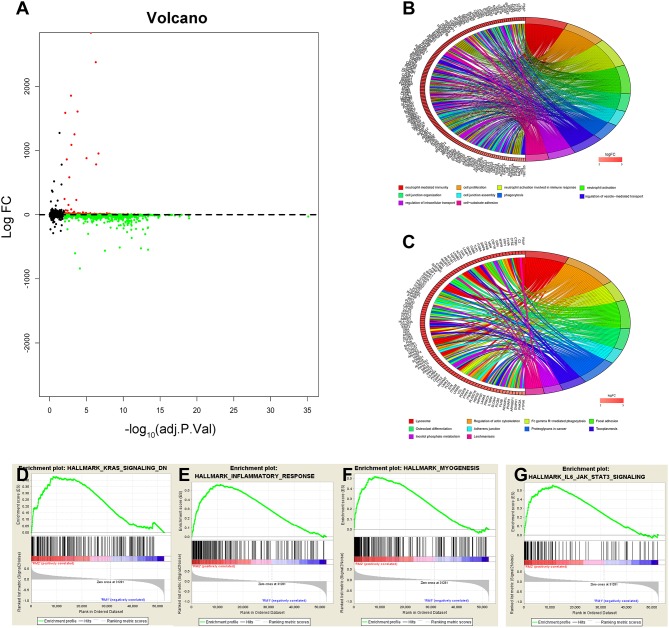
Interaction among m6A RNA methylation regulators and functional annotation of GBM in RM1/2 subgroups. **(A)** Volcano plot of identified DEGs. The red dots represent upregulated DEGs, and the green dots represent downregulated DEGs. **(B,C)** Functional annotation of the upregulated DEGs in the RM2 subgroup of GO analysis **(B)** and KEGG pathway analysis **(C)**. **(D–G)** GSEA revealed that genes with higher expression in the RM2 subgroup were enriched for hallmarks of malignant tumors.

The above results indicate that the consensus clustering results were closely related to the degree of malignancy of GBM. To better understand the mechanisms between the malignancy of GBM and the 13 m6A RNA methylation regulators, a total of 2,299 genes (599 genes were upregulated, and 1,700 genes were downregulated) were identified as DEGs by using differential analysis (Figure 3A). To summarize the potential function of DEGs, we performed an annotation of the 599 significantly upregulated genes (onco role, Table S3) in the RM2 subgroup through GO function analysis and KEGG pathway analysis. The top 10 GO terms indicated that upregulated genes were enriched in malignancy-related processes, including neutrophil-mediated immunity, cell proliferation, cell junction, phagocytosis, and cell-substrate adhesion (Figure 3B). KEGG pathway analysis top 10 terms testified that upregulated genes were related to the regulation of actin cytoskeleton pathway, focal adhesion pathway, proteoglycans in cancer pathway, and Fc gamma R-mediated phagocytosis pathway (Figure 3C). Furthermore, GSEA suggested that the malignant hallmarks of tumors, including KRAS signaling (NES = 1.59, normalized *P* = 0.013), inflammatory response (NES = 1.68, normalized *P* = 0.052), myogenesis (NES = 2.02, normalized *P* < 0.001), and IL-6/JAK/ STAT3 signaling (NES = 2.0, normalized *P* < 0.001), were significantly associated with the RM2 subgroup (Figures 3D–G). All these results proved that the two categories derived from consensus clustering analysis were closely related to the malignancy of GBM.

### Novel Candidate Compounds Targeting the Malignancy-Related Pathways and Biological Functions in GBM

**Figure 4:**
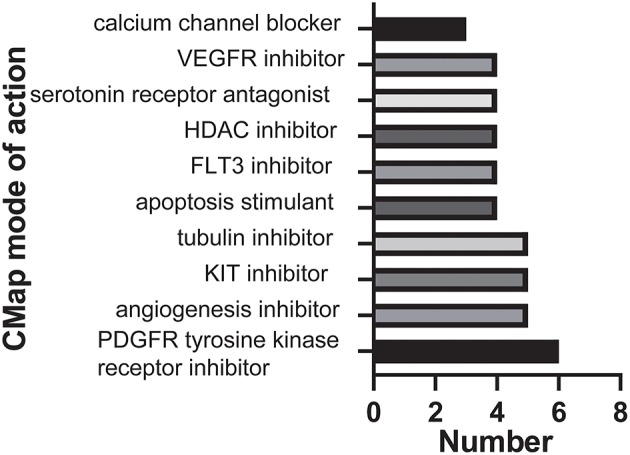
Histogram showing the number of compounds in the top 10 MoA, sorted by descending number of compounds with a shared MoA.

Next, we sought to determine the potential compounds that target malignancy-related pathways and biological functions; DEGs based on consensus clustering were submitted to retrieve the CMap databases (33). The top 89 compounds capable of repressing the above gene expression of GBM were summarized in Table S4 and Figure 4. One hundred seventy mechanisms were revealed through the CMap mode of action (MoA) analysis. Six compounds (Tandutinib, ENMD-2076, dasatinib, dovitinib, orantinib, tyrphostin-AG-1295) shared the MoA of PDGFR tyrosine kinase receptor inhibitor, and five compounds (ENMD-2076, mibefradil, NPI-2358, dovitinib, orantinib) shared the MoA of angiogenesis inhibitor. We also found that tandutinib, ENMD-2076, tivozanib, dasatinib, and dovitinib shared the MoA of KIT inhibitor.

### Identification and Analysis of m6A-Related Genes With Poor Prognosis in GBM

**Figure 5:**
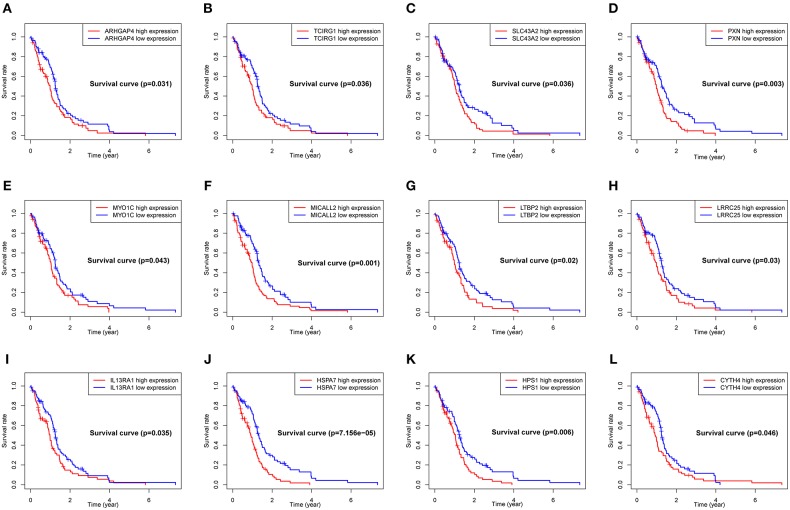
Correlation of expression of individual DEGs of OS in TCGA. Kaplan-Meier survival curves were generated for represented DEGs extracted from the comparison of groups of high (red line) and low (blue line) gene expression. **(A–L)** Representative Kaplan–Meier survival curves for selected DEGs. *p* < 0.05 in Log-rank test. OS, overall survival in years.

To explore the significance of each upregulated gene for the survival time of GBM patients from the TCGA database, Kaplan-Meier survival curves were generated. Among the 599 upregulated DEGs in the RM2 subgroup, a total of 79 DEGs (Table S3) were able to predict the poor OS in the log-rank test (*P* < 0.05, representative figures were shown in Figure 5).

**Figure 6:**
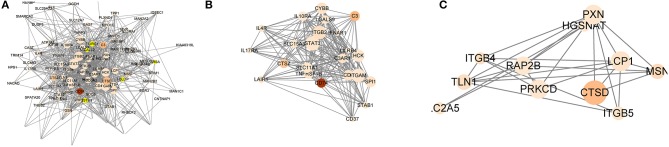
The integral and top-two networks of ITGAM and RAP2B modules. The color of a node in the PPI network reflects the type of interaction genes. (m6A RNA methylation regulators labeled as blue diamond, OS-related genes labeled as red circle). The size of the node indicates the number of interacting proteins with the designated protein. **(A–C)** The integral **(A)** and top-two PPI networks of ITGAM **(B)**, RAP2B **(C)** modules.

To better understand the interactions between the 79 genes with prognostic value and the 13 m6A RNA methylation regulators, we also analyzed the PPI among them using the STRING database. The network consists of four modules with a total of 88 nodes and 527 edges, which indicates that close interaction exists in this PPI network (Figure 6A). Among the 88 nodes, 54 central node genes (bold in Table S5) were selected with the filtering of degree > 10. The most significant 10 node degree genes were ITGAM, STAT3, SPI1, TNFRSF1B, MYO1F, SLC11A1, TCIRG1, RAP2B, FERMT3, and LCP1. The top two significant modules were selected by using the MCODE application for further analysis. To make the description more convenient, we named these modules “ITGAM module” and “RAP2B module,” respectively. Twenty-eight nodes and 165 edges were involved in ITGAM modules, with STAT3, SPI1, TNFRSF1B, SLC11A1, and FERMT3 being the remarkable nodes, as they had the most connections with other nodes in this module (Figure 6B). In the RAP2B module, 8 edges involving 6 nodes were formed in this network (Figure 6C). In addition, we predicted the function of the ITGAM module through GO analysis. It was related to the biological process of mRNA splicing via spliceosome, mRNA methylation, and oxidative single-stranded RNA demethylation. For instance, ITGAM was reported to play a critical role in invasive growth and angiogenesis in malignant gliomas (43). STAT3 methylation via STAT3 signaling could also promote tumorigenicity of GBM stem-like cells (44). The above results clearly demonstrate that m6A regulators participate in the critical malignant related biological regulatory network.

To find out whether the 79 OS-related DEGs found in the TCGA database were meaningful in the additional database, we further analyzed the expression profiles of 249 GBM cases from the CGGA database. Importantly, a total of 64 genes were validated to be significantly related to the poor prognosis, of which 37 genes (bold in Table S3) were of particular interest, as they have not been previously reported for their prognostic value in GBM patients (Figure S3).

### Prognostic Value of m6A RNA Methylation Regulators

**Figure 7:**
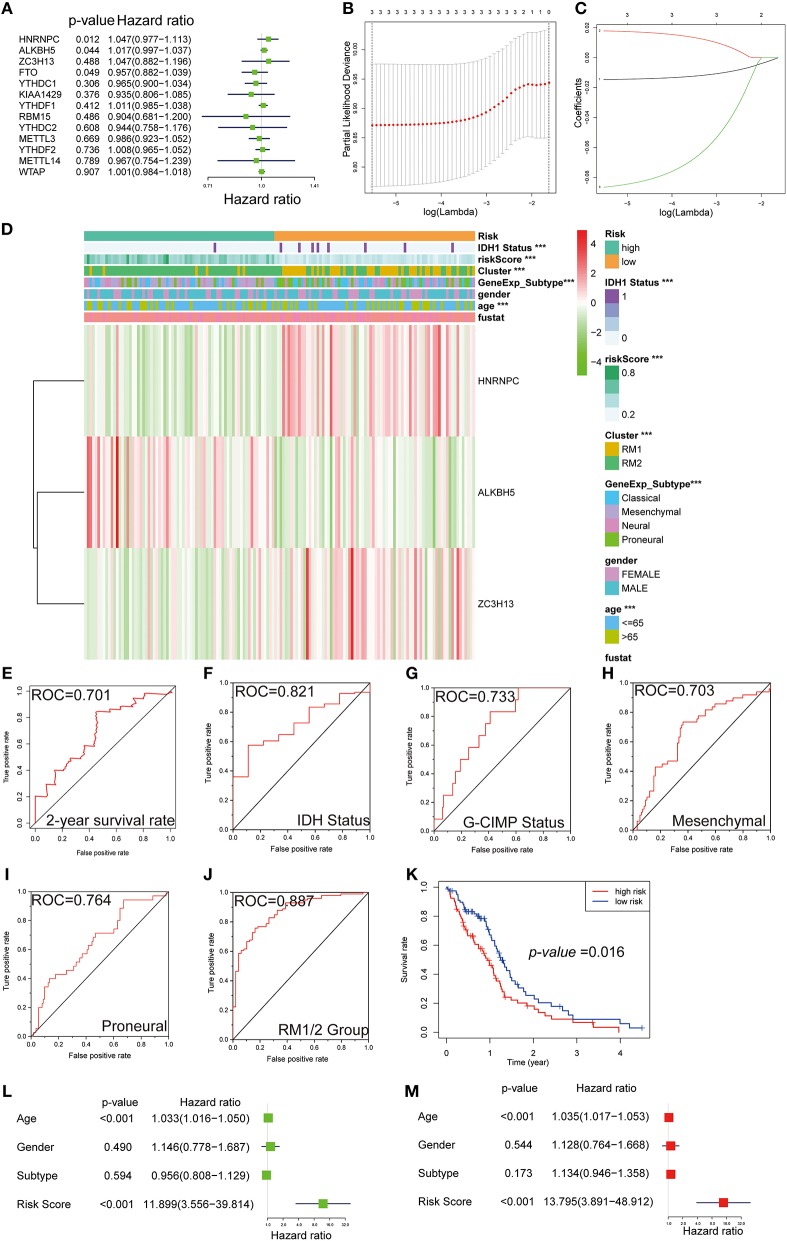
Gene signature with m6A RNA methylation regulators**. (A–C)** The process of building the signature containing three m6A RNA methylation regulators. The hazard ratios (HR) **(A)**, 95% confidence intervals (CI) calculated by univariate Cox regression, and the regression coefficients **(C)** calculated by multivariate Cox regression using LASSO **(B)** are exhibited. **(D)** The heatmap shows the expression levels of the three m6A RNA methylation regulators in low- and high-risk subgroups. The distribution of clinicopathological features was compared between the low- and high-risk groups. ***P < 0.001. **(E–J)** ROC curves show the predictive efficiency of the risk signature on the 2-year survival rate **(E)**, IDH status **(F)**, G-CIMP status **(G)**, mesenchymal subtype **(H)**, proneural subtype **(I)**, and RM1/2 subgroups **(J)**. **(K)** Kaplan–Meier survival curves for patients in the TCGA dataset assigned to high- and low-risk groups based on the risk score. **(L,M)** Univariate **(L)** and multivariate Cox regression analyses **(M)** of the association between clinicopathological factors (including the risk score) and OS of patients in the TCGA.

For the purpose of investigating the prognostic value of m6A RNA methylation regulators, univariate Cox regression analysis was performed on the expression profile data. Based on the information contained in these results, 4 of 13 genes exhibited a significant correlation with the prognosis. Among these four selected genes, HNRNPC, ALKBH5, and ZC3H13 were risky genes, with HR > 1, while FTO was a protective gene, with HR < 1 (Figure 7A).

Robust likelihood-based survival modeling and LASSO regression are widely used to screen prognostic genes in the context of high dimensional data and were therefore applied in our study. Compared with a single biomarker, integrating multiple biomarkers into one risk model could present a better prediction performance of the model. To remove the prediction errors and maintain the stability of the predictive prognosis, we specifically selected three genes (*P* < 0.05 and HR > 1) to develop the gene signature. Afterward, the above-selected genes with independent prognostic value, including NRNPC, ALKBH5, and ZC3H13, were screened as the candidate genes using LASSO regression. The regression coefficients based on the minimum criteria were used to assess the risk score for the GBM patients, and the coefficients of selected gene signatures were −0.014623, 0.017905, and −0.08661, respectively (Figures 7B,C).

### Gene-Signature Showed Strong Associations With Clinical Features in GBM

To investigate the prognostic value of the risk gene signature in the TCGA database, GBM patients were dichotomized into low- and high-risk subgroups, based on the median risk score. We next sought to detect the correlation between the two risk subgroups and clinical features—a heatmap was designed and showed the expression of the three selected m6A RNA methylation regulators (Figure 7D). Significant differences were observed in this heatmap between the high- and low-risk subgroups with respect to IDH1 status (*P* < 0.001), age (*P* < 0.001), molecular subtypes (*P* < 0.001), and RM1/2 subgroups (*P* < 0.001). To evaluate the predictive accuracy of the risk score model, we performed the ROC curve and calculated the AUC. The AUC was 0.701 in the 2-year ROC curve for the prognostic model (Figure 7E). For molecular phenotypes, such as IDH1 and G-CIMP status, the risk model also performed relatively well, with AUC was 0.821 and 0.733, respectively (Figures 7F,G). A similar trend was observed in subgroup analyses for mesenchymal and proneural, with AUCs of 0.703 and 0.764, respectively (Figures 7H,I). Moreover, the predicting power of the risk score model was significantly increased in RM1/2 subgroup analysis, with an AUC of 0.887 (Figure 7J). Notably, patients in the high-risk group exhibited significantly shorter survival time than those in the low-risk group (*P* < 0.05) (Figure 7H). Consistent with these findings, the patients with a high-risk score were also more sensitive to temozolomide chemotherapy, radiation therapy, and chemoradiation than low-risk score patients (Figure S4).

We further performed univariate and multivariate Cox proportional hazard regression analyses for the TCGA dataset to determine whether the risk signature was an independent prognostic factor. By univariate Cox analysis, age (HR = 1.033, *P* < 0.001) and risk score (HR = 11.899, *P* < 0.001) were all correlated with the OS, while GBM subtype and gender were not (Figure 7L). A similar trend of risk score was also observed when including these factors in the multivariate Cox proportional hazard regression (Figure 7M). The results demonstrated that age and risk score were independent prognostic factors in the TCGA GBM dataset. According to our results, the independent prognostic value and excellent prediction accuracy of the gene signature derived from the 13 m6A RNA methylation regulators were identified.

### Low Expression in Normal Brain Tissues of METTL3 and METTL14

**Figure 8:**
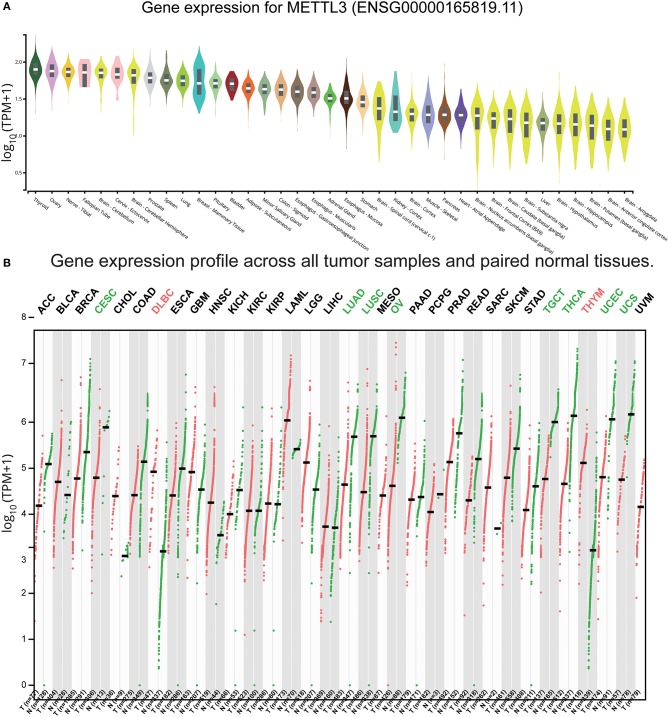
Expression of METTL3 in normal and GBM tissues. **(A)** The expression values of METTL3 of the different tissues in the organism. **(B)** The expression values of METTL3 in different tumors and corresponding normal tissues.

Based on our results and the evidence in literature, METTL3 was overexpressed specifically in GBM and was significantly related to the occurrence of GBM (45–47). To comprehensively understand the function of METTL3, we retrieved the expression levels of healthy tissue and tumor tissue in different parts from the GTEx and GEPIA databases (48), respectively. We found that the expression values of METTL3 in the brain were lower than other tissues in the organism (Figure 8A). Notably, the expression level of METTL3 in most tumors was smaller than the corresponding healthy tissue, except for GBM (Figure 8B). These results indicated that high expression of METTL3 might act as a driver of GBM and play a crucial role in GBM. Considering that METTL3 and METTL14 are both the most common and abundant mRNA modifications in eukaryotes, we also searched the expression profiles of METTL14 and found the same trend (Figure S5). The above results provided evidence for METTL3 and METTL14 as proto-oncogenes of GBM.

## Discussion

In the current study, we systematically analyzed the expression of m6A RNA regulators with different clinicopathological parameters and revealed the potential values. In particular, by comparing the expression of 13 regulators in a large number of healthy tissues and primary and recurrent tumor tissues, we found that they were related to the occurrence and recurrence of GBM. Furthermore, the expression of m6A RNA methylation regulators was also associated with the GBM subtype, G-CIMP status, and IDH status. Besides, GBM samples were classified into two subgroups, RM1/2, through consensus cluster analysis based on the expression of the 13 regulators. The RM1/2 subgroup not only affected OS and clinical characteristics, but also closely related to malignancy-related processes, key signaling pathways, and GBM hallmarks. Taking advantage of CMap, we also identified potential compounds targeting RNA methylation regulators in GBM. In addition, we obtained 79 genes with poor prognosis based on the RM1/2 subgroup by Kaplan–Meier analysis. Importantly, 64 genes with poor prognosis were validated in CGGA, a separate GBM database. Finally, we derived a prognostic gene signature, which dichotomized the OS of GBM patients into low- and high-risk subgroups and allowed us to extend the analysis. This risk gene-signature could be used as an independent prognostic marker and accurate clinicopathological parameters predictor.

Glioma was divided into GBM and low-grade glioma (LGG). GBM, as the most destructive glioma (WHO: IV), possesses significantly different genomics, treatment methods, clinical manifestations, characteristics, and prognosis from LGG (WHO: I–III) (49–52). Prior to this, the value of m6A methylation regulators in gliomas has been explored (53). Considering the comprehensive differences between LGG and GBM, we believed that this analysis was not sufficiently detailed and specific. Therefore, we specifically analyzed the specific value of these regulators in GBM. Similarly, we have all identified different hallmarks and pathways associated with malignancy. In particular, we further analyzed and obtained regulator-related specific targeted drugs and genes with poor prognosis, of which 37 genes have not been previously reported at the prognostic level. Especially, the PPI network between regulators and related genes was explored. Furthermore, we derived a prediction model that can predict the specific clinical characteristics and molecular phenotypes of GBM. Finally, we also provided evidence for the large transcriptome levels of METTL3 and METTL14 as cancer driver genes.

Among the m6A RNA methylation regulators, METTL3 or METTL14 is one of the most common and abundant mRNA modifications in eukaryotes. It has been reported that METTL3 or METTL14 inhibits the growth and self-renewal of the GBM stem cells (32). ALKBH5 was reported to maintain tumorigenicity of GBM stem cells by sustaining FOXM1 expression and cell proliferation program (54), suggesting a crucial tumorigenic role. Most recently, it has been reported that FTO plays a carcinogenic role through the FTO/m6A/MYC/CEBPA signaling pathway in IDH mutant cancers, such as glioma and leukemia (19, 55). The differences of involved genes among different tumor types give us a clue that altered the expression of key genes, which are sensitive to the function of m6A methylation regulators, can cause significant phenotype changes.

In this section, we rounded analyzed the expression of all m6A RNA methylation regulators in GBM at the occurrence and recurrence stages. Unlike our study, a previous trial showed that ALKBH5 was an oncogene to maintain tumorigenicity, while our study showed a significantly decreased trend in the GBM group compared with normal. However, unlike the previous trial, our study included a large number of clinical samples and was validated in two databases. This difference in the number of samples may account for the different results. Interestingly, the upward trend in ALKBH5 was significantly associated with tumor recurrence when we compared primary and recurrent tumors. It's worth mentioning that ALKBH5 belongs to the AlkB family of non-heme Fe(II)/a-ketoglutarate-dependent dioxygenases, and the activity is iron-dependent (17). Given our results, we further speculate that iron metabolism is involved in GBM recurrence (56, 57). However, this hypothesis needs to be more tested. Nevertheless, a tendency toward a lower expression of FTO was observed in GBM compared with normal tissues. Unlike ALKBH5, FTO was found to mediate the demethylation of m6Am instead of m6A preferentially. It seems that FTO and ALKBH5 mediate the demethylation of different methylation targets in GBM, which is worthy of future research. Based on this difference in demethylation targets and tendencies, we speculate that ALKBH5 and FTO have different functions and mechanisms in GBM and are worthy of further study.

Nearly all IDH-mutant GBMs harbored G-CIMP and patients carrying G-CIMP (G-CIMP+) have been confirmed to confer a better clinical outcome than those not carrying (G-CIMP–) (58). Collectively, we conclude that the expression of m6A RNA methylation regulators is closely associated with the occurrence, recurrence, IDH status, G-CIMP status, and molecular subtype of GBM. Moreover, these findings of the expression of each individual m6A methylation regulator can contribute to the development of new cancer therapies, as chemotherapy targeting m6A methylation is now at the forefront of cancer therapy.

We demonstrated that m6A RNA methylation regulators were also related to the biological processes, cellular component, and signaling pathways of GBM malignant progression. RNA m6A methylation is a nascent field as of yet, the significance of the above epigenetics marker in human cancer is just beginning to be appreciated. Although the m6A modification showed tissue-specific regulation and increased significantly throughout the brain development process (3), studies (59, 60) on the role of m6A modification in either brain lesions or brain cancers have only been reported sporadically (61, 62). Several biological processes and signaling pathways have already been identified: tumor stem-like cell regulation, including maintenance, radio-resistance, and tumorigenesis; post-transcriptional modification, including in RNA transcript, RNA processing, RNA processing, RNA degradation, and RNA translation; FTO/m6A/MYC/CEBPA signaling pathways (19); JAK1/STAT5/C/EBP β pathways (63); and the IL-7/STAT5/SOCS pathways (64). This report provided the potential biological process and pathway between RNA m6A methylation and GBM-malignant progression, which represent a significant step toward developing therapeutic strategies to treat GBM by targeting m6A modification.

CMap can identify biomarkers for predicting specific drug reactions, mechanisms of treatment, and ways to overcome them (65–67). CMap analysis, which is based on a limited number of treated cell lines, accurately identified a number of compounds that have been shown to have an effect on m6A of other tumor types with specificity (33, 68–70). METTL3 has been reported to promote gastric cancer angiogenesis by secreting HDGF (71). This result verified the accuracy of our CMap-based drug prediction from the side. Based on these results, hence, we speculated that PDGFR tyrosine kinase receptor inhibitor, KIT inhibitor, and tubulin inhibitor could all be used as potential agents that specifically target m6A-related biological functions and pathways for subsequent research.

METTL3, served as a methyltransferase, has been reported to be essential for glioma stem-like cell maintenance and radio-resistance (45). Our findings further confirm that METTL3 was a potential therapeutic target, and future research is expected to focus on studies that specifically target METTL3. Since the small-molecule inhibitors of METTL3 have not yet been invented, future research should focus on this area (72).

This study identified and validated that 64 genes were associated with poor outcomes in GBM patients. Moreover, we were able to construct four PPI modules, all of which were related to critical GBM biological processes. Highly relevant nodes in the modules, including STAT3, SLC11A1, and ITGAM, have been reported to promote tumor proliferation, angiogenesis, migration, and invasiveness (73–77). Among the 64 genes validated, 27 (such as ALOX5, CAST, HS6ST1, ITGAM, PTPN6, SLC11A1, and SLC12A7) have been reported to be involved in the pathogenesis of GBM or critical in predicting OS. This suggests that our big data-based analyses using TCGA and CGGA cohorts harbor predictive value. Although the remaining 37 genes have not been previously reported to be associated with GBM prognosis, they can be used as a potential clinical prognostic indicator for GBM patients, which can facilitate clinicians to make more accurate diagnosis easily.

In this study, we attempted to introduce some concepts associated with the theory of the prognosis value of m6A RNA methylation regulators based on uncovered sets. METTL3 has been reported as a potential biomarker panel for prognostic prediction in colorectal carcinoma (78). The prognostic model of multiple m6A RNA methylation regulators for patients with GBM has not been developed. The GBM prognostic gene signature based on three selected m6A RNA methylation regulators was designed for the first time. As we observed, the risk score calculated by the correlation coefficient conferred the ability in prognosis and clinicopathological parameter prediction. Excitingly, Cox analysis results further confirmed the independent prognostic value of the risk score. Meanwhile, GBM patients with a high-risk score showed more sensitivity to temozolomide chemotherapy, radiation therapy, and chemoradiation than low-risk-score patients. These findings may deepen our understanding of m6A methylation regulators in prognosis level and tolerance to chemoradiotherapy.

To sum up, we attempted to identify the expressions, potential functions, and prognostic values of m6A RNA methylation regulators in GBM. Our study provides strategies for comprehensive analysis of cancer genomics based on consensus clustering analysis for systematic identification of specific m6A-related targets and specific targeted drugs based on m6A RNA methylation regulators. The prognostic gene signature and genes with poor prognosis might contribute to the personalized prediction of GBM prognosis and serve as a potential biomarker reflecting GBM patients' response to therapies that specifically target m6A. Finally, further investigation of these genes could lead to novel insights into the potential association of m6A methylation regulators with GBM prognosis in a comprehensive manner.

## Data Availability Statement

Publicly available datasets were analyzed in this study. This data can be found here: https://portal.gdc.cancer.gov; http://www.cgga.org.cn; http://commonfund.nih.gov/GTEx/.

## Author Contributions

JD, RX, LC, and SH conceived and designed the study and drafted the manuscript. JD and KH collected, analyzed, and interpreted the data. HJ, SMi, YB, and SMa participated in revising the manuscript. All authors have read and approved the final manuscript.

### Conflict of Interest

The authors declare that the research was conducted in the absence of any commercial or financial relationships that could be construed as a potential conflict of interest.

## References

[1] Machnicka MA Milanowska K Osman Oglou O Purta E Kurkowska M Olchowik A. MODOMICS: a database of RNA modification pathways−2013 update. Nucleic Acids Res. (2013) 41:D262–7. 10.1093/nar/gks1007 23118484 PMC3531130

[2] Su Y Huang J Hu J. m(6)A RNA methylation regulators contribute to malignant progression and have clinical prognostic impact in gastric cancer. Front Oncol. (2019) 9:1038. 10.3389/fonc.2019.01038 31681576 PMC6813557

[3] Meyer KD Saletore Y Zumbo P Elemento O Mason CE Jaffrey SR. Comprehensive analysis of mRNA methylation reveals enrichment in 3' UTRs and near stop codons. Cell. (2012) 149:1635–46. 10.1016/j.cell.2012.05.003 22608085 PMC3383396

[4] Wei CM Gershowitz A Moss B. Methylated nucleotides block 5' terminus of HeLa cell messenger RNA. Cell. (1975) 4:379–86. 10.1016/0092-8674(75)90158-0 164293

[5] Dominissini D Moshitch-Moshkovitz S Schwartz S Salmon-Divon M Ungar L Osenberg S. Topology of the human and mouse m6A RNA methylomes revealed by m6A-seq. Nature. (2012) 485:201–6. 10.1038/nature11112 22575960

[6] Schumann U Shafik A Preiss T. METTL3 Gains R/W access to the epitranscriptome. Mol Cell. (2016) 62:323–4. 10.1016/j.molcel.2016.04.024 27153530

[7] Liu J Yue Y Han D Wang X Fu Y Zhang L. A METTL3-METTL14 complex mediates mammalian nuclear RNA N6-adenosine methylation. Nat Chem Biol. (2014) 10:93–5. 10.1038/nchembio.1432 24316715 PMC3911877

[8] Ping XL Sun BF Wang L Xiao W Yang X Wang WJ. Mammalian WTAP is a regulatory subunit of the RNA N6-methyladenosine methyltransferase. Cell Res. (2014) 24:177–89. 10.1038/cr.2014.3 24407421 PMC3915904

[9] Meyer KD Jaffrey SR. Rethinking m(6)A readers, writers, and erasers. Annu Rev Cell Dev Biol. (2017) 33:319–42. 10.1146/annurev-cellbio-100616-060758 28759256 PMC5963928

[10] Schwartz S Mumbach MR Jovanovic M Wang T Maciag K Bushkin GG. Perturbation of m6A writers reveals two distinct classes of mRNA methylation at internal and 5' sites. Cell Rep. (2014) 8:284–96. 10.1016/j.celrep.2014.05.048 24981863 PMC4142486

[11] Wen J Lv R Ma H Shen H He C Wang J. Zc3h13 regulates nuclear RNA m(6)A methylation and mouse embryonic stem cell self-renewal. Mol Cell. (2018) 69:1028–38 e6. 10.1016/j.molcel.2018.02.015 29547716 PMC5858226

[12] Haussmann IU Bodi Z Sanchez-Moran E Mongan NP Archer N Fray RG. m(6)A potentiates Sxl alternative pre-mRNA splicing for robust drosophila sex determination. Nature. (2016) 540:301–4. 10.1038/nature20577 27919081

[13] Patil DP Pickering BF Jaffrey SR. Reading m(6)A in the transcriptome: m(6)A-binding proteins. Trends Cell Biol. (2018) 28:113–27. 10.1016/j.tcb.2017.10.001 29103884 PMC5794650

[14] Zhao BS Roundtree IA He C. Publisher correction: post-transcriptional gene regulation by mRNA modifications. Nat Rev Mol Cell Biol. (2018) 19:808. 10.1038/s41580-018-0075-1 30341428

[15] Jia G Fu Y Zhao X Dai Q Zheng G Yang Y. N6-methyladenosine in nuclear RNA is a major substrate of the obesity-associated FTO. Nat Chem Biol. (2011) 7:885–7. 10.1038/nchembio.687 22002720 PMC3218240

[16] Li Y Yang F Gao M Gong R Jin M Liu T. miR-149–3p Regulates the switch between adipogenic and osteogenic differentiation of BMSCs by targeting FTO. Mol Ther Nucleic Acids. (2019) 17:590–600. 10.1016/j.omtn.2019.06.023 31382190 PMC6690430

[17] Zheng G Dahl JA Niu Y Fedorcsak P Huang CM Li CJ. ALKBH5 is a mammalian RNA demethylase that impacts RNA metabolism and mouse fertility. Mol Cell. (2013) 49:18–29. 10.1016/j.molcel.2012.10.015 23177736 PMC3646334

[18] Barbieri I Tzelepis K Pandolfini L Shi J Millan-Zambrano G Robson SC. Promoter-bound METTL3 maintains myeloid leukaemia by m(6)A-dependent translation control. Nature. (2017) 552:126–31. 10.1038/nature24678 29186125 PMC6217924

[19] Su R Dong L Li C Nachtergaele S Wunderlich M Qing Y. R-2HG exhibits anti-tumor activity by targeting FTO/m(6)A/MYC/CEBPA signaling. Cell. (2018) 172:90–105 e23. 10.1016/j.cell.2017.11.031 29249359 PMC5766423

[20] Ma JZ Yang F Zhou CC Liu F Yuan JH Wang F. METTL14 suppresses the metastatic potential of hepatocellular carcinoma by modulating N(6) -methyladenosine-dependent primary MicroRNA processing. Hepatology. (2017) 65:529–43. 10.1002/hep.28885 27774652

[21] Bartosovic M Molares HC Gregorova P Hrossova D Kudla G Vanacova S. N6-methyladenosine demethylase FTO targets pre-mRNAs and regulates alternative splicing and 3'-end processing. Nucleic Acids Res. (2017) 45:11356–70. 10.1093/nar/gkx778 28977517 PMC5737695

[22] Zhao X Yang Y Sun BF Shi Y Yang X Xiao W. FTO-dependent demethylation of N6-methyladenosine regulates mRNA splicing and is required for adipogenesis. Cell Res. (2014) 24:1403–19. 10.1038/cr.2014.151 25412662 PMC4260349

[23] Geula S Moshitch-Moshkovitz S Dominissini D Mansour AA Kol N Salmon-Divon M. Stem cells. m6A mRNA methylation facilitates resolution of naive pluripotency toward differentiation. Science. (2015) 347:1002–6. 10.1126/science.1261417 25569111

[24] Wang X Lu Z Gomez A Hon GC Yue Y Han D. N6-methyladenosine-dependent regulation of messenger RNA stability. Nature. (2014) 505:117–20. 10.1038/nature12730 24284625 PMC3877715

[25] Wang X Zhao BS Roundtree IA Lu Z Han D Ma H. N(6)-methyladenosine modulates messenger RNA translation efficiency. Cell. (2015) 161:1388–99. 10.1016/j.cell.2015.05.014 26046440 PMC4825696

[26] Xiang Y Laurent B Hsu CH Nachtergaele S Lu Z Sheng W. RNA m(6)A methylation regulates the ultraviolet-induced DNA damage response. Nature. (2017) 543:573–6. 10.1038/nature21671 28297716 PMC5490984

[27] Vu LP Pickering BF Cheng Y Zaccara S Nguyen D Minuesa G. The N(6)-methyladenosine. (m(6)A)-forming enzyme METTL3 controls myeloid differentiation of normal hematopoietic and leukemia cells. Nat Med. (2017) 23:1369–76. 10.1038/nm.4416 28920958 PMC5677536

[28] Liu J Eckert MA Harada BT Liu SM Lu Z Yu K. m(6)A mRNA methylation regulates AKT activity to promote the proliferation and tumorigenicity of endometrial cancer. Nat Cell Biol. (2018) 20:1074–83. 10.1038/s41556-018-0174-4 30154548 PMC6245953

[29] Stupp R Hegi ME Mason WP van den Bent MJ Taphoorn MJ Janzer RC. Effects of radiotherapy with concomitant and adjuvant temozolomide versus radiotherapy alone on survival in glioblastoma in a randomised phase III study: 5-year analysis of the EORTC-NCIC trial. Lancet Oncol. (2009) 10:459–66. 10.1016/S1470-2045(09)70025-7 19269895

[30] Chen X Zhao C Guo B Zhao Z Wang H Fang Z. Systematic profiling of alternative mRNA splicing signature for predicting glioblastoma prognosis. Front Oncol. (2019) 9:928. 10.3389/fonc.2019.00928 31608231 PMC6769083

[31] Wang Y Liu X Guan G Xiao Z Zhao W Zhuang M. Identification of a five-pseudogene signature for predicting survival and its ceRNA network in glioma. Front Oncol. (2019) 9:1059. 10.3389/fonc.2019.01059 31681595 PMC6803554

[32] Cui Q Shi H Ye P Li L Qu Q Sun G. m(6)A RNA methylation regulates the self-renewal and tumorigenesis of glioblastoma stem cells. Cell Rep. (2017) 18:2622–34. 10.1016/j.celrep.2017.02.059 28297667 PMC5479356

[33] Subramanian A Narayan R Corsello SM Peck DD Natoli TE Lu X. A next generation connectivity map: l1000 platform and the first 1,000,000 profiles. Cell. (2017) 171:1437–52 e17. 10.1016/j.cell.2017.10.049 29195078 PMC5990023

[34] Wang Q Armenia J Zhang C Penson AV Reznik E Zhang L. Unifying cancer and normal RNA sequencing data from different sources. Sci Data. (2018) 5:180061. 10.1038/sdata.2018.61 29664468 PMC5903355

[35] Wilkerson MD Hayes DN. ConsensusClusterPlus: a class discovery tool with confidence assessments and item tracking. Bioinformatics. (2010) 26:1572–3. 10.1093/bioinformatics/btq170 20427518 PMC2881355

[36] Ritchie ME Phipson B Wu D Hu Y Law CW Shi W. limma powers differential expression analyses for RNA-sequencing and microarray studies. Nucleic Acids Res. (2015) 43:e47. 10.1093/nar/gkv007 25605792 PMC4402510

[37] Subramanian A Tamayo P Mootha VK Mukherjee S Ebert BL Gillette MA. Gene set enrichment analysis: a knowledge-based approach for interpreting genome-wide expression profiles. Proc Natl Acad Sci USA. (2005) 102:15545–50. 10.1073/pnas.0506580102 16199517 PMC1239896

[38] Franceschini A Szklarczyk D Frankild S Kuhn M Simonovic M Roth A. STRING v9.1: protein-protein interaction networks, with increased coverage and integration. Nucleic Acids Res. (2013) 41:D808–15. 10.1093/nar/gks1094 23203871 PMC3531103

[39] Shannon P Markiel A Ozier O Baliga NS Wang JT Ramage D. Cytoscape: a software environment for integrated models of biomolecular interaction networks. Genome Res. (2003) 13:2498–504. 10.1101/gr.1239303 14597658 PMC403769

[40] Zhang Y Li H Zhang W Che Y Bai W Huang G. LASSObased CoxPH model identifies an 11lncRNA signature for prognosis prediction in gastric cancer. Mol Med Rep. (2018) 18:5579–93. 10.3892/mmr.2018.9567 30365077 PMC6236314

[41] Goeman JJ. L1 penalized estimation in the cox proportional hazards model. Biom J. (2010) 52:70–84. 10.1002/bimj.200900028 19937997

[42] Verhaak RG Hoadley KA Purdom E Wang V Qi Y Wilkerson MD. Integrated genomic analysis identifies clinically relevant subtypes of glioblastoma characterized by abnormalities in PDGFRA, IDH1, EGFR, and NF1. Cancer Cell. (2010) 17:98–110. 10.1016/j.ccr.2009.12.020 20129251 PMC2818769

[43] Schnell O Krebs B Wagner E Romagna A Beer AJ Grau SJ. Expression of integrin alphavbeta3 in gliomas correlates with tumor grade and is not restricted to tumor vasculature. Brain Pathol. (2008) 18:378–86. 10.1111/j.1750-3639.2008.00137.x 18394009 PMC2607528

[44] Kim E Kim M Woo DH Shin Y Shin J Chang N. Phosphorylation of EZH2 activates STAT3 signaling via STAT3 methylation and promotes tumorigenicity of glioblastoma stem-like cells. Cancer Cell. (2013) 23:839–52. 10.1016/j.ccr.2013.04.008 23684459 PMC4109796

[45] Visvanathan A Patil V Arora A Hegde AS Arivazhagan A Santosh V. Essential role of METTL3-mediated m(6)A modification in glioma stem-like cells maintenance and radioresistance. Oncogene. (2018) 37:522–33. 10.1038/onc.2017.351 28991227

[46] Visvanathan A Patil V Abdulla S Hoheisel JD Somasundaram K N(6)-Methyladenosine landscape of glioma stem-like cells: METTL3 is essential for the expression of actively transcribed genes and sustenance of the oncogenic signaling. Genes. (2019) 10:141 10.3390/genes10020141 PMC6410051 30781903

[47] Tuncel G Kalkan R. Importance of m N(6)-methyladenosine. (m(6)A) RNA modification in cancer. Med Oncol. (2019) 36:36. 10.1007/s12032-019-1260-6 30879160

[48] Tang Z Li C Kang B Gao G Li C Zhang Z. GEPIA: a web server for cancer and normal gene expression profiling and interactive analyses. Nucleic Acids Res. (2017) 45:W98–102. 10.1093/nar/gkx247 28407145 PMC5570223

[49] Jakola AS Myrmel KS Kloster R Torp SH Lindal S Unsgard G. Comparison of a strategy favoring early surgical resection vs a strategy favoring watchful waiting in low-grade gliomas. JAMA. (2012) 308:1881–8. 10.1001/jama.2012.12807 23099483

[50] Van Meir EG Hadjipanayis CG Norden AD Shu HK Wen PY Olson JJ. Exciting new advances in neuro-oncology: the avenue to a cure for malignant glioma. CA Cancer J Clin. (2010) 60:166–93. 10.3322/caac.20069 20445000 PMC2888474

[51] Perry JR Laperriere N Mason WP Radiation plus temozolomide in patients with glioblastoma. N Engl J Med. (2017) 376:2197 10.1056/NEJMoa1611977 28564572

[52] Cancer Genome Atlas Research N Brat DJ Verhaak RG Aldape KD Yung WK Salama SR. Comprehensive, integrative genomic analysis of diffuse lower-grade gliomas. N Engl J Med. (2015) 372:2481–98. 10.1056/NEJMoa1402121 26061751 PMC4530011

[53] Chai RC Wu F Wang QX Zhang S Zhang KN Liu YQ. m(6)A RNA methylation regulators contribute to malignant progression and have clinical prognostic impact in gliomas. Aging. (2019) 11:1204–25. 10.18632/aging.101829 30810537 PMC6402513

[54] Zhang S Zhao BS Zhou A Lin K Zheng S Lu Z. m(6)A demethylase ALKBH5 maintains tumorigenicity of glioblastoma stem-like cells by sustaining FOXM1 expression and cell proliferation program. Cancer Cell. (2017) 31:591–606 e6. 10.1016/j.ccell.2017.02.013 28344040 PMC5427719

[55] Li Z Weng H Su R Weng X Zuo Z Li C. FTO plays an oncogenic role in acute myeloid leukemia as a N(6)-methyladenosine RNA demethylase. Cancer Cell. (2017) 31:127–41. 10.1016/j.ccell.2016.11.017 28017614 PMC5234852

[56] Legendre C Garcion E. Iron metabolism: a double-edged sword in the resistance of glioblastoma to therapies. Trends Endocrinol Metab. (2015) 26:322–31. 10.1016/j.tem.2015.03.008 25936466

[57] Schoenfeld JD Sibenaller ZA Mapuskar KA Wagner BA Cramer-Morales KL Furqan M. O2(-) and H2O2-mediated disruption of fe metabolism causes the differential susceptibility of NSCLC and GBM cancer cells to pharmacological ascorbate. Cancer Cell. (2017) 31:487–500 e8. 10.1016/j.ccell.2017.07.008 28366679 PMC5497844

[58] Malta TM de Souza CF Sabedot TS Silva TC Mosella MS Kalkanis SN. Glioma CpG island methylator phenotype. (G-CIMP): biological and clinical implications. Neuro Oncol. (2018) 20:608–20. 10.1093/neuonc/nox183 29036500 PMC5892155

[59] Hibar DP Stein JL Renteria ME Arias-Vasquez A Desrivieres S Jahanshad N. Common genetic variants influence human subcortical brain structures. Nature. (2015) 520:224–9. 10.1038/nature14101 25607358 PMC4393366

[60] Yoon KJ Ringeling FR Vissers C Jacob F Pokrass M Jimenez-Cyrus D. Temporal control of mammalian cortical neurogenesis by m(6)A methylation. Cell. (2017) 171:877–89 e17. 10.1016/j.cell.2017.09.003 28965759 PMC5679435

[61] Lin X Chai G Wu Y Li J Chen F Liu J. RNA m(6)A methylation regulates the epithelial mesenchymal transition of cancer cells and translation of snail. Nat Commun. (2019) 10:2065. 10.1038/s41467-019-09865-9 31061416 PMC6502834

[62] Paris J Morgan M Campos J Spencer GJ Shmakova A Ivanova I. Targeting the RNA m(6)A reader YTHDF2 selectively compromises cancer stem cells in acute myeloid leukemia. Cell Stem Cell. (2019) 25:137–48 e6. 10.1016/j.stem.2019.03.021 31031138 PMC6617387

[63] Yao Y Bi Z Wu R Zhao Y Liu Y Liu Q. METTL3 inhibits BMSC adipogenic differentiation by targeting the JAK1/STAT5/C/EBPbeta pathway via an m(6)A-YTHDF2-dependent manner. FASEB J. (2019) 33:7529–44. 10.1096/fj.201802644R 30865855

[64] Li HB Tong J Zhu S Batista PJ Duffy EE Zhao J. m(6)A mRNA methylation controls T cell homeostasis by targeting the IL-7/STAT5/SOCS pathways. Nature. (2017) 548:338–42. 10.1038/nature23450 28792938 PMC5729908

[65] Petrich AM Leshchenko V Kuo PY Xia B Thirukonda VK Ulahannan N. Akt inhibitors MK-2206 and nelfinavir overcome mTOR inhibitor resistance in diffuse large B-cell lymphoma. Clin Cancer Res. (2012) 18:2534–44. 10.1158/1078-0432.CCR-11-1407 22338016 PMC3889476

[66] Leshchenko VV Kuo PY Jiang Z Thirukonda VK Parekh S. Integrative genomic analysis of temozolomide resistance in diffuse large B-cell lymphoma. Clin Cancer Res. (2014) 20:382–92. 10.1158/1078-0432.CCR-13-0669 24178621

[67] Xiong DD Xu WQ He RQ Dang YW Chen G Luo DZ. *In silico* analysis identified miRNAbased therapeutic agents against glioblastoma multiforme. Oncol Rep. (2019) 41:2194–208. 10.3892/or.2019.7022 30816530 PMC6412522

[68] Brum AM van de Peppel J Nguyen L Aliev A Schreuders-Koedam M Gajadien T. Using the connectivity map to discover compounds influencing human osteoblast differentiation. J Cell Physiol. (2018) 233:4895–906. 10.1002/jcp.26298 29194609

[69] Lian H Han YP Zhang YC Zhao Y Yan S Li QF. Integrative analysis of gene expression and DNA methylation through one-class logistic regression machine learning identifies stemness features in medulloblastoma. Mol Oncol. (2019) 13:2227–45. 10.1002/1878-0261.12557 31385424 PMC6763787

[70] Malta TM Sokolov A Gentles AJ Burzykowski T Poisson L Weinstein JN. Machine learning identifies stemness features associated with oncogenic dedifferentiation. Cell. (2018) 173:338–54 e15. 10.1016/j.cell.2018.03.034 29625051 PMC5902191

[71] Wang Q Chen C Ding Q Zhao Y Wang Z Chen J METTL3-mediated m(6)A modification of HDGF mRNA promotes gastric cancer progression and has prognostic significance. Gut. (2019). 10.1136/gutjnl-2019-319639. [Epub ahead of print]. 31582403

[72] Lan Q Liu PY Haase J Bell JL Huttelmaier S Liu T. The critical role of RNA m(6)A methylation in cancer. Cancer Res. (2019) 79:1285–92. 10.1158/0008-5472.CAN-18-2965 30894375

[73] Wu P Cai J Chen Q Han B Meng X Li Y. Lnc-TALC promotes O(6)-methylguanine-DNA methyltransferase expression via regulating the c-Met pathway by competitively binding with miR-20b-3p. Nat Commun. (2019) 10:2045. 10.1038/s41467-019-10025-2 31053733 PMC6499807

[74] Jiang J Zhang L Chen H Lei Y Zhang T Wang Y. Regorafenib induces lethal autophagy arrest by stabilizing PSAT1 in glioblastoma. Autophagy. (2019) 16:106–22. 10.1080/15548627.2019.1598752 30909789 PMC6984601

[75] Maximov V Chen Z Wei Y Robinson MH Herting CJ Shanmugam NS. Tumour-associated macrophages exhibit anti-tumoural properties in sonic hedgehog medulloblastoma. Nat Commun. (2019) 10:2410. 10.1038/s41467-019-10458-9 31160587 PMC6546707

[76] Takenaka MC Gabriely G Rothhammer V Mascanfroni ID Wheeler MA Chao CC. Control of tumor-associated macrophages and T cells in glioblastoma via AHR and CD39. Nat Neurosci. (2019) 22:729–40. 10.1038/s41593-019-0370-y 30962630 PMC8052632

[77] Xie B Zhang L Hu W Fan M Jiang N Duan Y. Dual blockage of STAT3 and ERK1/2 eliminates radioresistant GBM cells. Redox Biol. (2019) 24:101189. 10.1016/j.redox.2019.101189 30986607 PMC6463934

[78] Li T Hu PS Zuo Z Lin JF Li X Wu QN. METTL3 facilitates tumor progression via an m(6)A-IGF2BP2-dependent mechanism in colorectal carcinoma. Mol Cancer. (2019) 18:112. 10.1186/s12943-019-1038-7 31230592 PMC6589893

