# Supplementary material for: Malignant Evaluation and Clinical Prognostic Values of m6A RNA Methylation Regulators in Glioblastoma
Source: Front Oncol. 2020 Mar 9;10:208. doi: 10.3389/fonc.2020.00208 (PMC7075451; doi:10.3389/fonc.2020.00208)
Supplement: Supplementary file 1 [file Data_Sheet_1.docx]

Supplementary Material

## Supplementary Figures


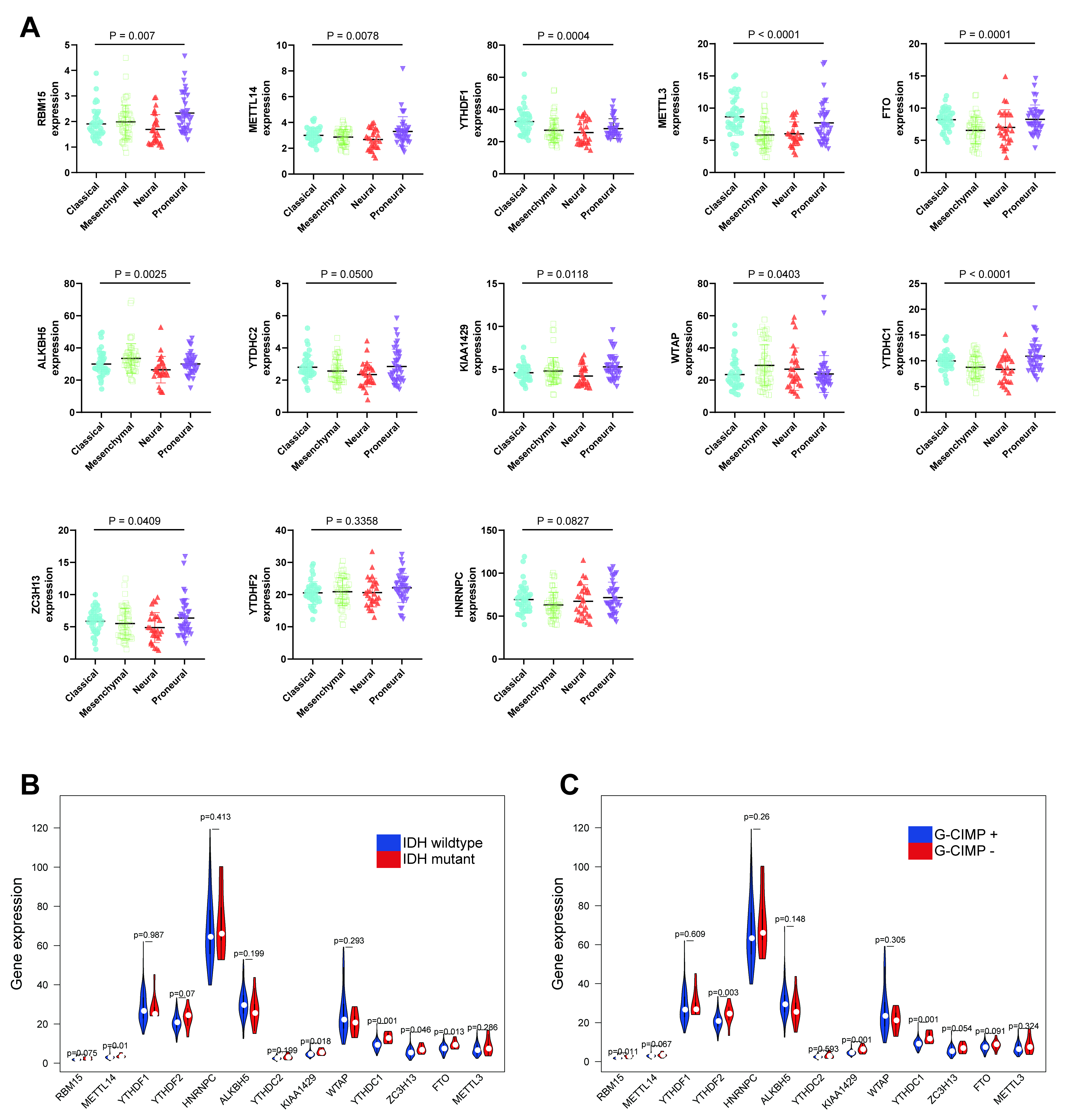


**Figure S1. Expression pattern of m6A RNA methylation regulators in the molecular phenotype of GBM**. (**A-C**) The relationship between m6A RNA methylation regulators expression level and GBM subtypes (Classical, Mesenchymal, Neural, Proneural) (**A**), IDH status(**B**), G-CIMP status (**C**).


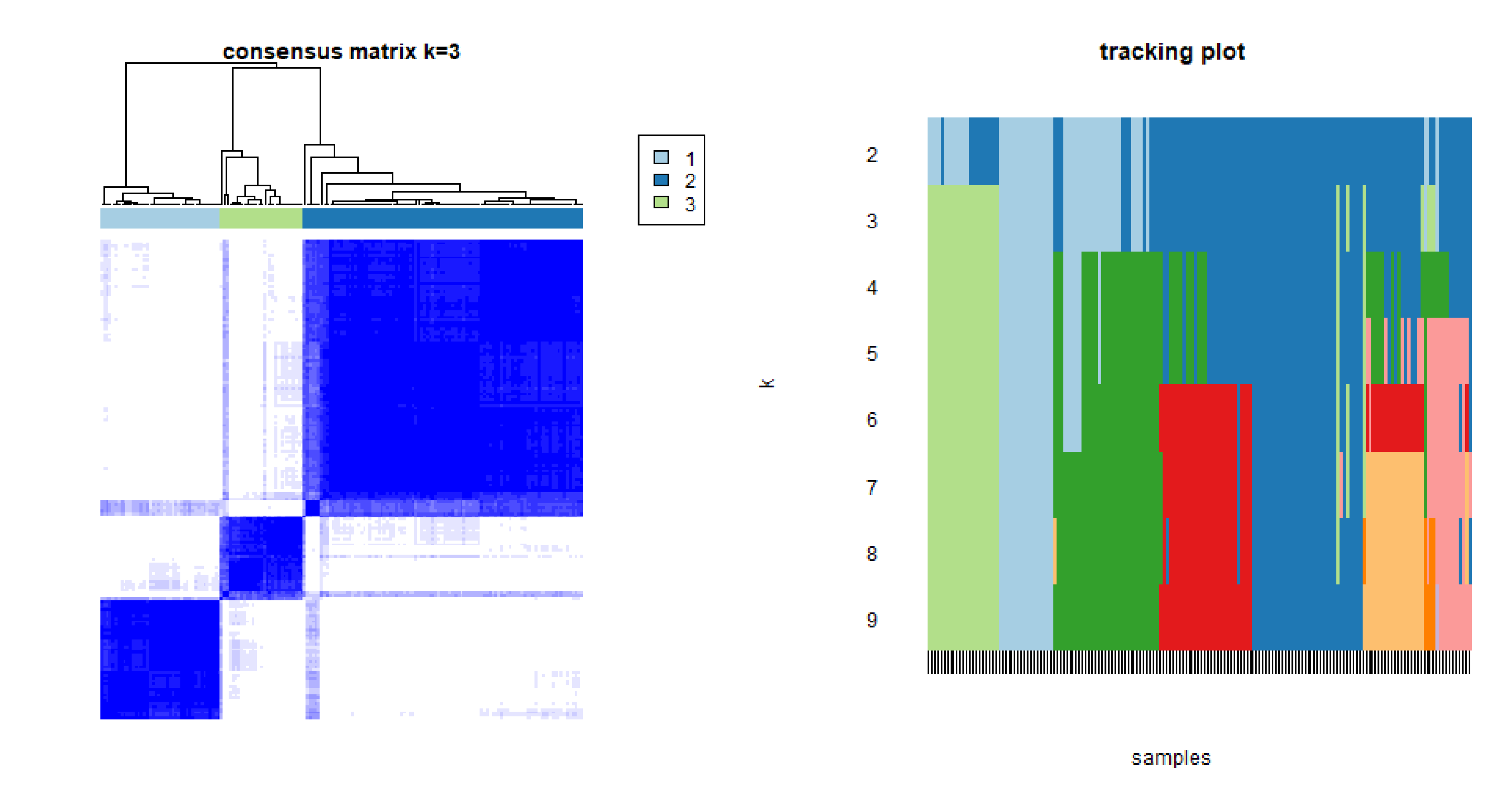


**Figure S2. Identification of consensus clusters by m6A RNA methylation regulators.** (**A**) Consensus clustering matrix for k = 3, (**B**) The tracking plot for k=2 to k=10.


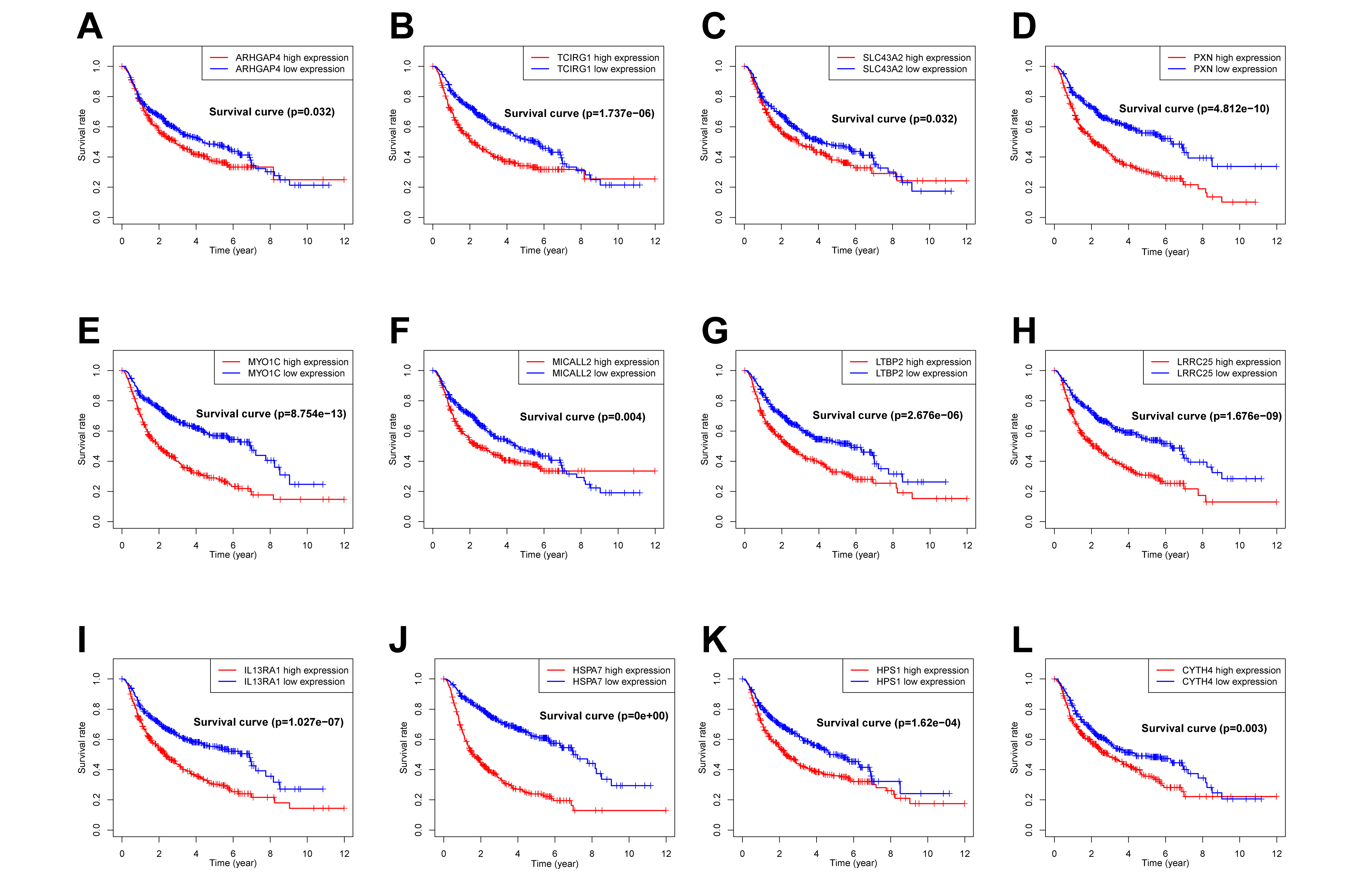


**Figure S3. Validation of correlation of DEGs extracted from the TCGA database with overall survival in the CGGA cohort.** Kaplan-Meier survival curves were generated for selected DEGs extracted from the comparison of groups of high (red line) and low (blue line) gene expression.


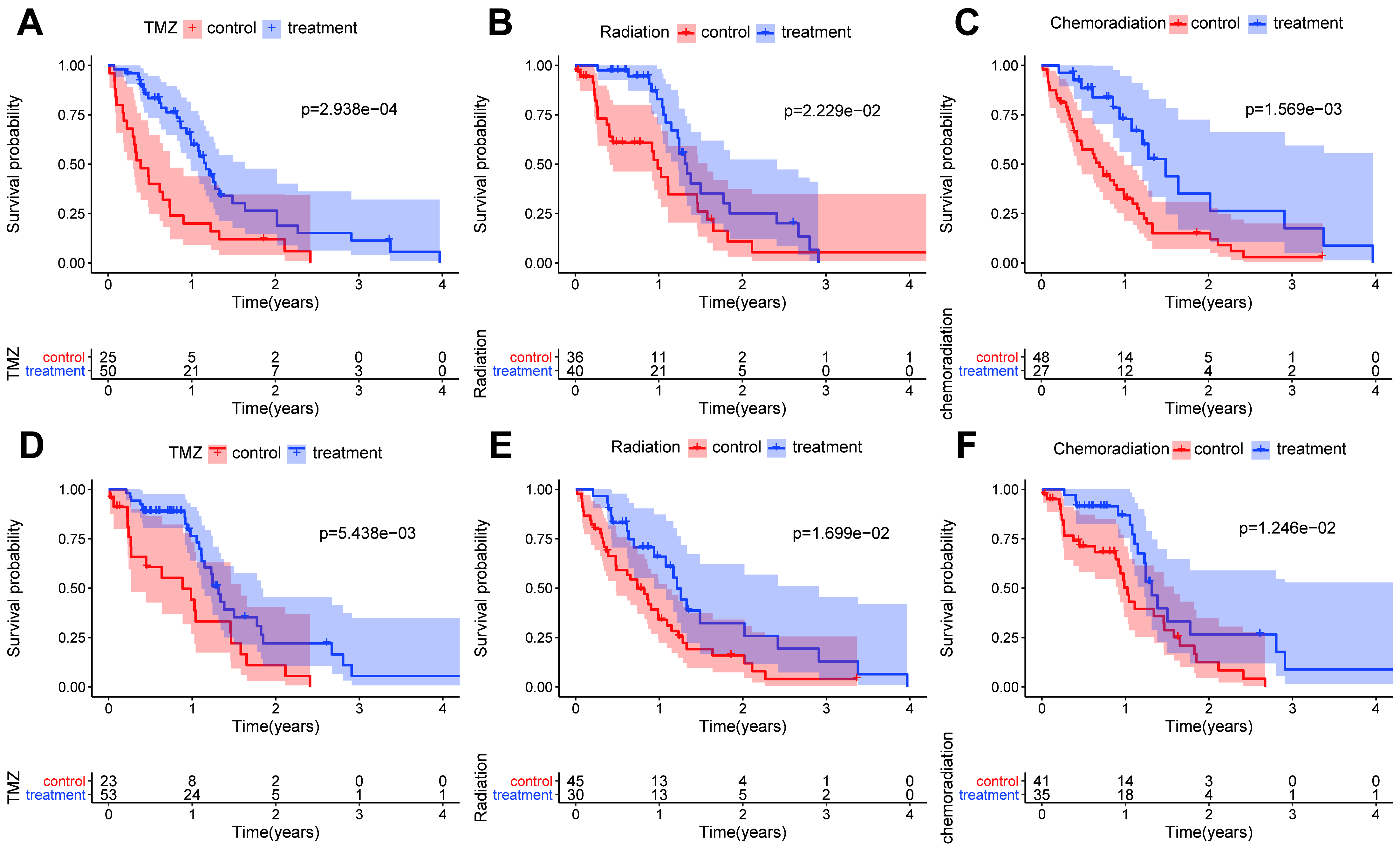


**Figure S4. Kaplan–Meier survival curves for high- and low-risk groups of different clinical therapies**. (A-C) Kaplan–Meier survival curves for high-risk group GBM patients with temozolomide chemotherapy (A), radiation therapy (B), and chemoradiation (C). (D-F) Kaplan–Meier survival curves for high-risk group GBM patients with temozolomide chemotherapy (D), radiation therapy (E), and chemoradiation (F).


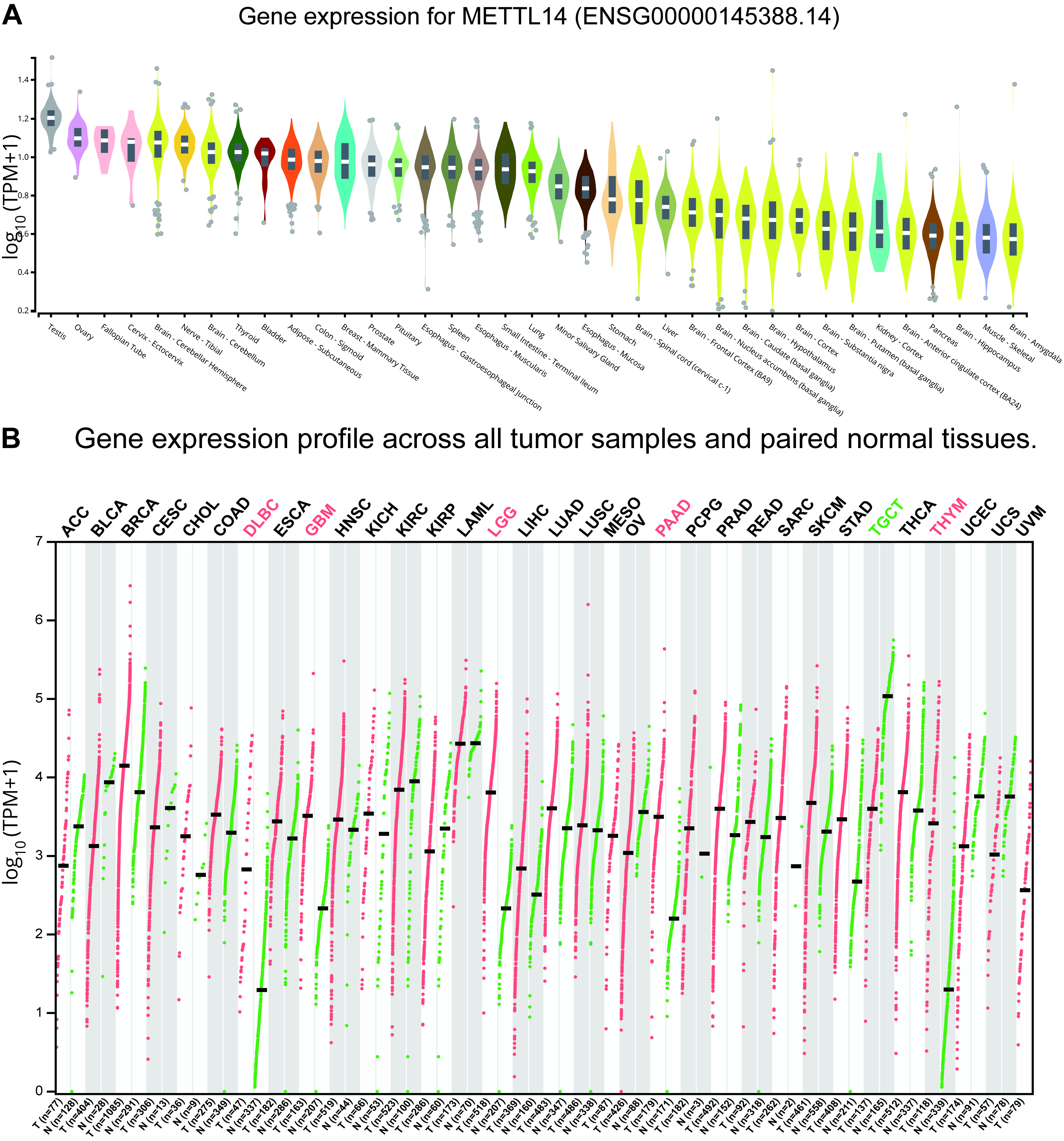


**Figure S5. Expression of METTL14 in normal and GBM tissues**. (**A**) The expression values of METTL14 of the different tissues in the organism. (**B**) The expression values of METTL14 in different tumors and corresponding normal tissues.
