# Supplementary material for: Malignant Evaluation and Clinical Prognostic Values of m6A RNA Methylation Regulators in Glioblastoma
Source: Front Oncol. 2020 Mar 9;10:208. doi: 10.3389/fonc.2020.00208 (PMC7075451; doi:10.3389/fonc.2020.00208)
Supplement: Supplementary file 2 [file Table_1.DOCX]

**Table S1. Clinicopathological features of patients included in this study.**

|  |  | TCGA dataset | | CGGA dataset | |
| --- | --- | --- | --- | --- | --- |
|  |  | Number | Percentage | Number | Percentage |
| Total |  | 174 | 100% | 354 | 100% |
| N/T |  |  |  |  |  |
|  | Norma | 5 | 2.87% | 105 | 29.66% |
|  | Tumor | 169 | 97.12% | 249 | 70.34% |
| Gender |  |  |  |  |  |
|  | Female | 59 | 34.91% | 102 | 40.96% |
|  | Male | 110 | 65.08% | 147 | 59.04% |
| Subtype |  |  |  |  |  |
|  | Classical | 42 | 25.45% | NG |  |
|  | Mesenchymal | 56 | 33.94% | NG |  |
|  | Neural | 28 | 16.97% | NG |  |
|  | Proneural | 39 | 23.64% | NG |  |
| P/R |  |  |  |  |  |
|  | Primary | 156 | 92.31% | 140 | 56.22% |
|  | Recurrence | 13 | 7.69% | 109 | 43.78% |
| Fustat |  |  |  |  |  |
|  | Dead | 118 | 69.82% | 166 | 77.21% |
|  | Alive | 51 | 30.18% | 49 | 21.79% |
| Age |  |  |  |  |  |
|  | <=65 | 109 | 64.50 % | 221 | 88.76% |
|  | >65 | 60 | 35.50 % | 28 | 11.24% |

(105 healthy samples showed in CGGA dataset were obtained From GTEx database )
