# Supplementary material for: Malignant Evaluation and Clinical Prognostic Values of m6A RNA Methylation Regulators in Glioblastoma
Source: Front Oncol. 2020 Mar 9;10:208. doi: 10.3389/fonc.2020.00208 (PMC7075451; doi:10.3389/fonc.2020.00208)
Supplement: Supplementary file 3 [file Table_2.DOCX]

**Table S2. Clinicopathological features of patients between RM1 and RM2.**

|  |  | RM1 | RM2 | P-value |
| --- | --- | --- | --- | --- |
| Total cases |  | 52 | 106 |  |
| Gender |  |  |  | 0.133 |
|  | Female | 24 | 32 |  |
|  | Male | 28 | 74 |  |
| Subtype |  |  |  | 6.639E-03 |
|  | Classical | 12 | 27 |  |
|  | Mesenchymal | 12 | 41 |  |
|  | Neural | 11 | 17 |  |
|  | Proneural | 17 | 21 |  |
| Age |  |  |  | 4.593E-02 |
|  | <=65 | 33 | 70 |  |
|  | >65 | 19 | 36 |  |
| Fustat |  |  |  | 0.447 |
|  | Dead | 31 | 79 |  |
|  | Alive | 21 | 27 |  |
